# MACHINE LEARNING APPROACHES TO ENHANCE CLAIMS DATA ANALYSES

**DOI:** 10.1093/geroni/igz038.1596

**Published:** 2019-11-08

**Authors:** Ricardo Pietrobon, David Marcozzi

**Affiliations:** 1 SporeData Inc, Durham, North Carolina, United States; 2 University of Maryland, Baltimore, Maryland, United States

## Abstract

This presentation will cover recent advances in machine learning applied to large claims databases involving medical disparities. First, we will describe methods involving the enrichment of existing claims data with social determinants of health from census data, where variables are imputed from one dataset to another, ultimately resulting in clinical models with enhanced predictive performance. Second, we will discuss the inclusion of variables representing imaging signs from MRI and CT exams, presenting large scalability and interobserver reliability, representing a method that can be used to enrich large state and national registries through the use of image recognition. Finally, we will discuss novel protocols for Natural Language Processing involving a combination of rule-based creation of corpora for radiology and discharge reports, with highly accurate deep learning methods for concept extraction and classification.

